# Complete genome sequences of seven *Microbacterium foliorum* phages Albedo, Kenzers, Swervy, Cranjis, JaimeB, Fullmetal, and Stormbreaker

**DOI:** 10.1128/mra.01251-23

**Published:** 2024-03-07

**Authors:** Keyshla Valentin Caban, Elizabeth Kalesnik, Kaitlyn A. Green, Christopher J. Negro, Ulises Nunez Rodriguez, Milan C. Peele, Casandra T. Nguyen, Sydney Cahill, Keara Dougherty, Melissa Logue, Star Hargraves, Hannah Radziak, Luke Willette, Esther Ogunyinka, Davia C. Campbell, Oluwatomiwa Adebamiro, Cecelia Schmeltzer, Jonathan Onimus, Hannah A. Asaka, Wilfred Bangura, Christina M. Shimp, Ameera Alade, Daekwon M. Sequira, Tommy Jimenez, Neumann University Phage Discovery Group, Sarah J. Swerdlow, Melinda K. Harrison, Patricia C. Fallest-Strobl, Matthew D. Mastropaolo

**Affiliations:** 1 Sciences, Neumann University, Aston, Pennsylvania, USA; 2 Biological Sciences, University of Pittsburgh Greensburg, Greensburg, Pennsylvania, USA; 3 Sciences, Cabrini University, Radnor, Pennsylvania, USA; DOE Joint Genome Institute, USA

**Keywords:** bacteriophages, genomics, cluster, DNA sequencing

## Abstract

Seven bacteriophages were isolated from soil in Pennsylvania and Wisconsin using the host *Microbacterium foliorum*. These bacteriophages range in the number of predicted genes encoded, from 25 to 91, and are distributed across actinobacteriophage clusters EB, EC, EE, and EK.

## ANNOUNCEMENT

Bacteriophages are incredibly abundant and genetically diverse. To expand our knowledge of bacteriophage evolution and diversity, we report here the characteristics of seven bacteriophages newly isolated using *Microbacterium foliorum* NRRL B-24224 (1, 2).

All seven bacteriophages were isolated from soil in Pennsylvania and Wisconsin using standard methods as previously described (Table 1) (3, 4). These soil samples were incubated in peptone-yeast extract-calcium (PYCa) liquid medium for 2 hours at 30°C with shaking to suspend phage particles. The suspension was then filtered through a 0.22-µm filter. The filtrate was either directly plated in PYCa soft agar containing *M. foliorum* or “enriched” by inoculation with *M. foliorum* and incubation at 30°C for 2–3 days before being filtered and plated (Table 1), yielding phages Albedo, Kenzers, Swervy, Cranjis, JaimeB, Fullmetal, and Stormbreaker. All phages were purified through three rounds of plating. All plates were incubated at 30°C for 24–48 hours.

**TABLE 1:** Bacteriophage, plaque morphology, and genomic characteristics

Phage name	Soil sample collection site	Isolation method	Plaque morphology	Plaque Size^*a*^ (mm)	Approx. shotgun coverage (fold)	No. of 150-bp single-end reads	Genome length (bp)	Genome end characteristic	G + C content (%)	No. of ORFs^*b*^	No. of tRNAs	Cluster^*c*^
Albedo	Hudson, WI, 44.984533 N, 92.7545 W	Enriched	Clear with halo^*d*^	0.1–1	83	862,002	41,813	3′ single-stranded overhang 5′-TCTCCCGGCA-3′	66.6	71	1 (Gln)	EB
Kenzers	Greenville, PA, 41.4124 N, 80.3813 W	Enriched	Clear	1	3,293	960,944	41,261	3′ single-stranded overhang 5′-TCTCCCGGCA-3′	66.8	70	1 (Gln)	EB
Swervy	Aston, PA, 39.8657 N, 75.4279 W	Direct	Turbid	0.5–1	325	93,814	41,510	3′ single-stranded overhang 5′-TCTCCCGGCA-3′	66.7	71	1 (Asn)	EB
Cranjis	Upper Chichester, PA, 39.856232 N, 75.443149 W	Direct	Turbid	3.5–4	99	40,843	53,222	Circularly permutated	68.9	91	0	EC
JaimeB	Aston, PA, 39.875331 N, 75.440021 W	Direct	Clear	3–4	14,635	1.8 million	17,445	3′ single-stranded overhang 5′-CCCGCCCCA-3′	68.7	25	0	EE
Stormbreaker	Aston, PA, 39.5215 N, 75.260806 W	Direct	Clear	1	1,073	74,440	54,050	Circularly permutated	60	54	0	EK^*e*^
Fullmetal	Aston, PA, 39.876667 N, 75.441667 W	Direct	Clear	1–1.5	197	410,171	54,438	Circularly permutated	59.8	55	0	EK^*f*^

^*a*^ Plaque size is based on the measurements of three plaques.

^*b*^ ORFs, open reading frames.

^*c*^ Clusters were identified using sequence similarities to other *Microbacterium* phage (5).

^*d*^ Indicates a clear middle of the plaque with a diffuse or cloudy edge.

^*e*^ Subcluster EK2.

^*f*^ Subcluster EK1.

^*g*^ "ND” indicates that the TEM was not performed.

^*h*^ Isolation methods are described in the Phage Discovery Guide (3, 4).

The Wizard DNA Cleanup Kit (Promega) was used to extract genomic DNA from phage lysates, as previously described (4). Some lysates were concentrated using ZnCl_2_ precipitation prior to genomic DNA extraction (6). The genomic DNA libraries were prepared using a NEBNext Ultra II FS Kit (New England BioLabs) followed by sequencing using Illumina MiSeq (v3 reagents), yielding at least 40,000 150-base single-end reads (Table 1). Raw reads were assembled and then checked for completeness using Newbler v2.9 (7) and Consed v29 (8), respectively (9). Sequencing results and genome characteristics of each bacteriophage are listed in Table 1.

The genomes were autoannotated using DNA Master v5.23.6 (http://cobamide2.bio.pitt.edu), Glimmer v3.02b (10), GeneMark v4.28 (11) and were refined using PECAAN v20221109 (https://pecaan.kbrinsgd.org/index.html), Starterator v462 (https://github.com/SEA-PHAGES/starterator), and Phamerator v539 (12). Transmembrane helices were predicted using SOSUI v1.11 (13), TOPCONS v2.0 (14), TMHMM v2.0 (15), and DeepTMHMM v1.0.24 (16). tRNAs were predicted using ARAGORN v1.2.41 (17) and tRNAscanSE v2.0 (18). Putative functions for other predicted genes were made using HHPRED v3.2 (against the PDB_mmCIF70, NCBI_Conserved_Domains, Pfam-A, and UniProt-SwissProt databases) (19) and BlastP v2.10.0 (against the PhagesDB and NCBI nonredundant databases) (20). All annotations were performed with default parameters.

Phages were assigned to clusters based on gene content similarity (GCS) of at least 35% to sequenced genomes in the Acinobacteriophage database (https://phagesdb.org/) using the GCS tool at phagesDB (5, 21). All seven phages reported here are consistent with features previously described for their respective clusters; the EB cluster phages, Albedo, Kenzers, and Swervy encode for <3 tRNAs; the EC cluster phage Cranjis has all its genes transcribed rightward; the EE cluster phage JaimeB shares all 25 predicted genes including a capsid maturation and protease fusion protein with the other EE cluster members; the EK cluster phages Stormbreaker and Fullmetal have the first ~30 predicted genes transcribed leftward and all the remaining genes transcribed rightward, and they also encode for the largest actinobacteriophage gene product, over 4,400 amino acids (1, 22).

## Data Availability

All genomes, Albedo, Kenzers, Swervy, Cranjis, JaimeB, Fullmetal, and Stormbreaker are available at GenBank with Accession No. OR475283, OP172875, MZ747513, OP297543, OR195050, OP297538, MT657334 and the Sequence Read Archive (SRA) No. SRX22868877, SRX14483228, SRX14485092, SRX22853654, SRX22853656, SRX22853655, SRX22853658, respectively.
